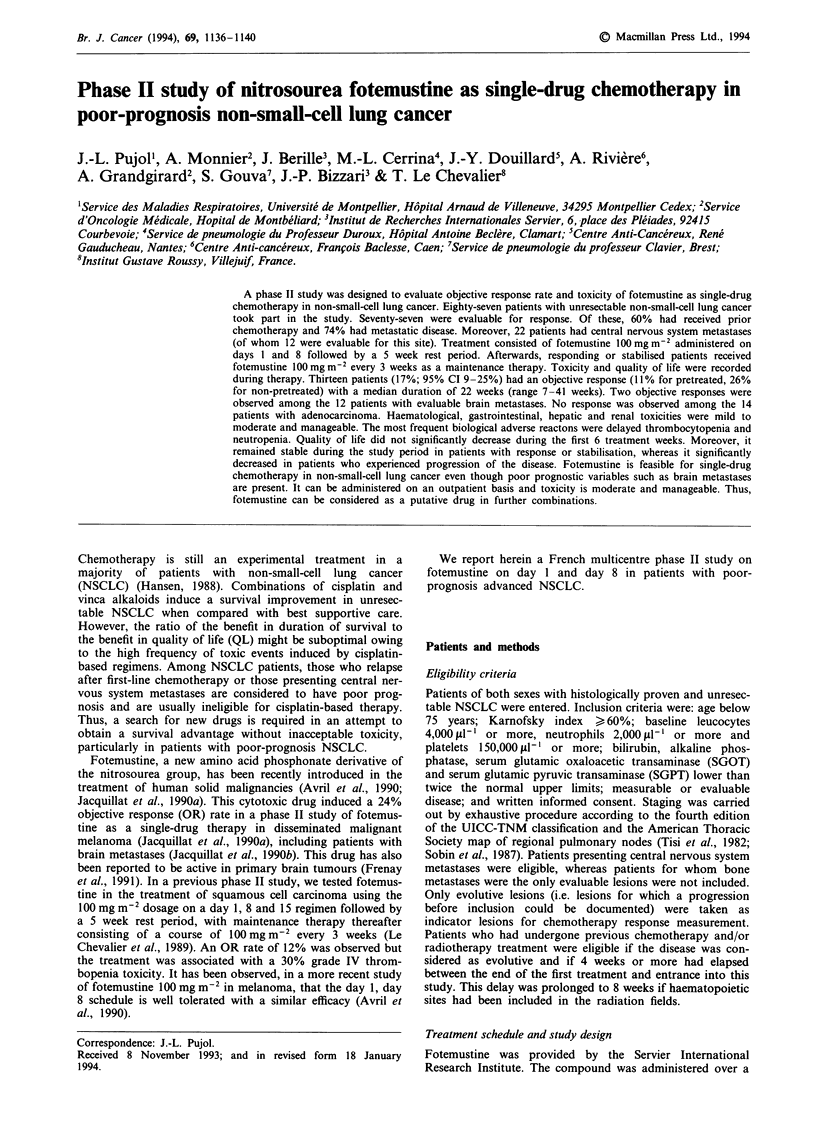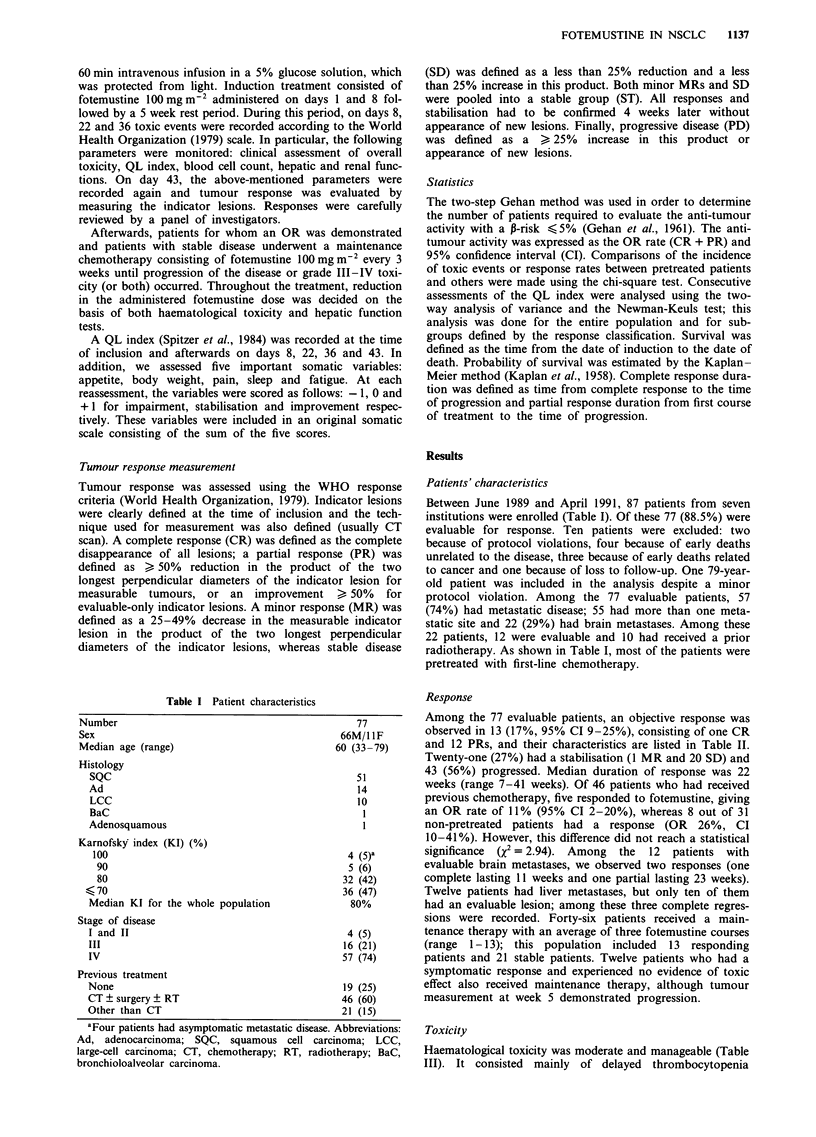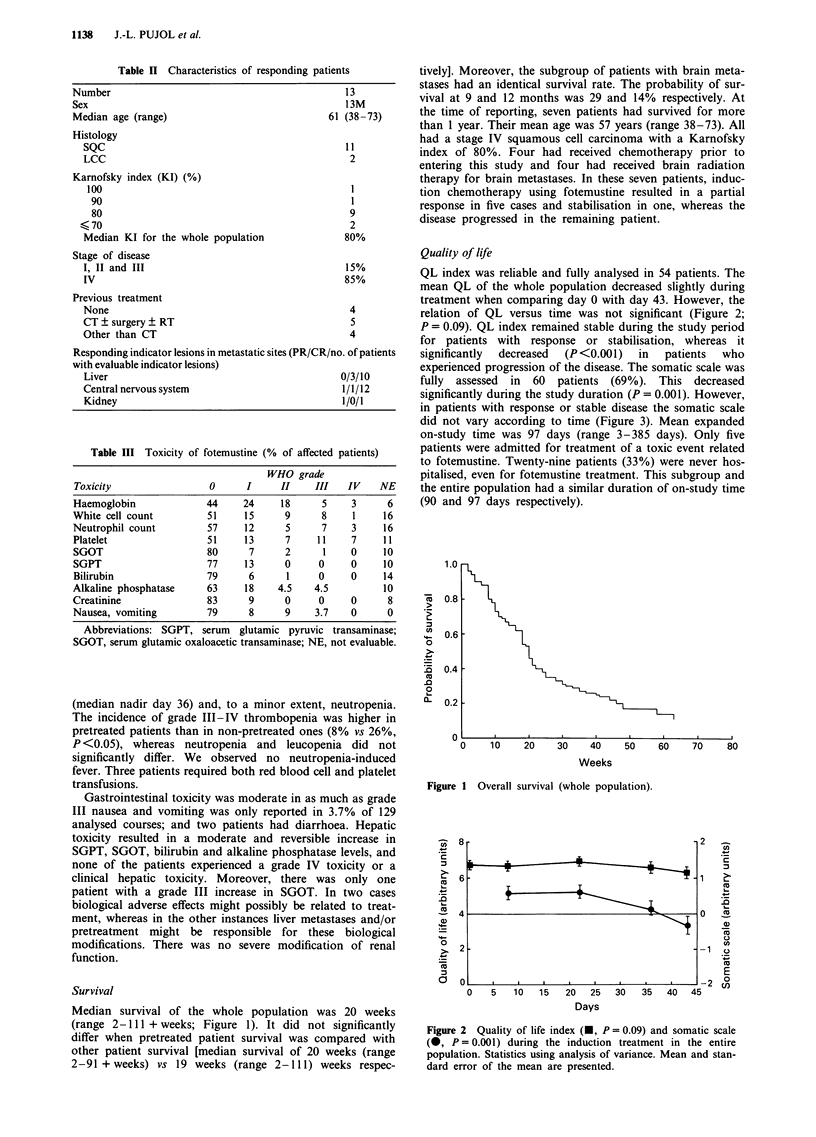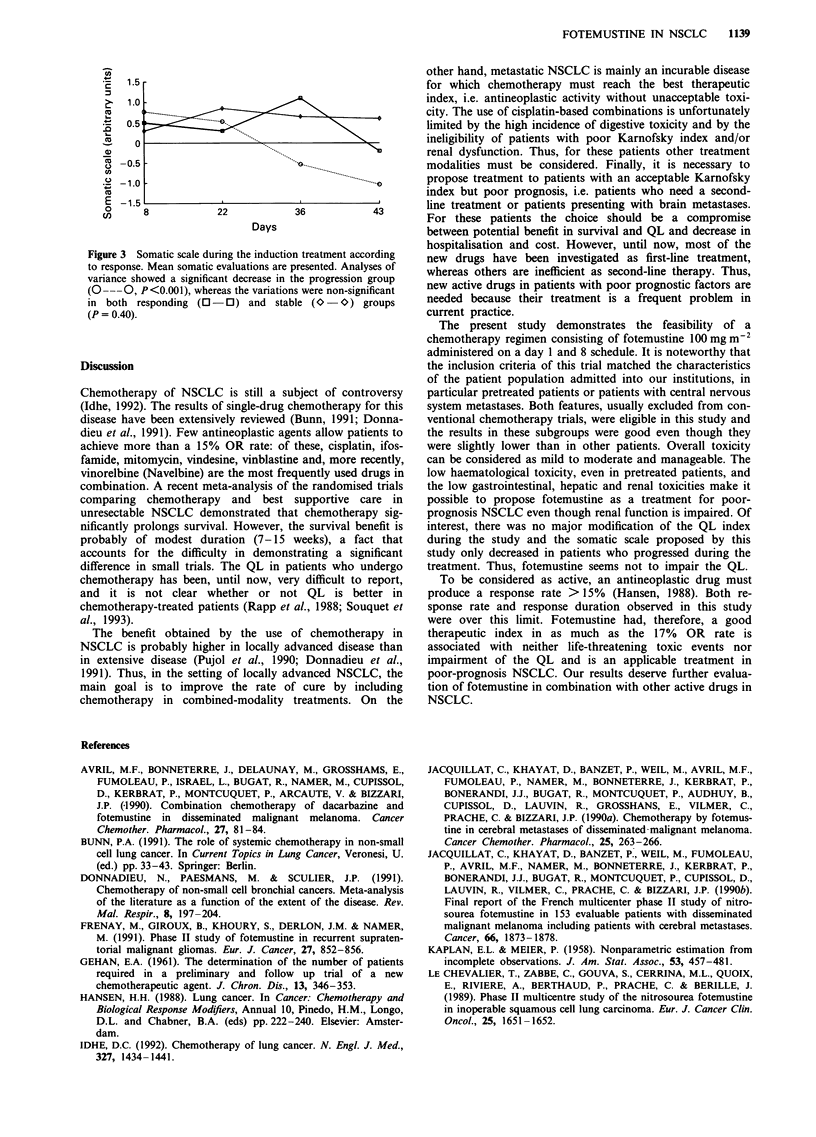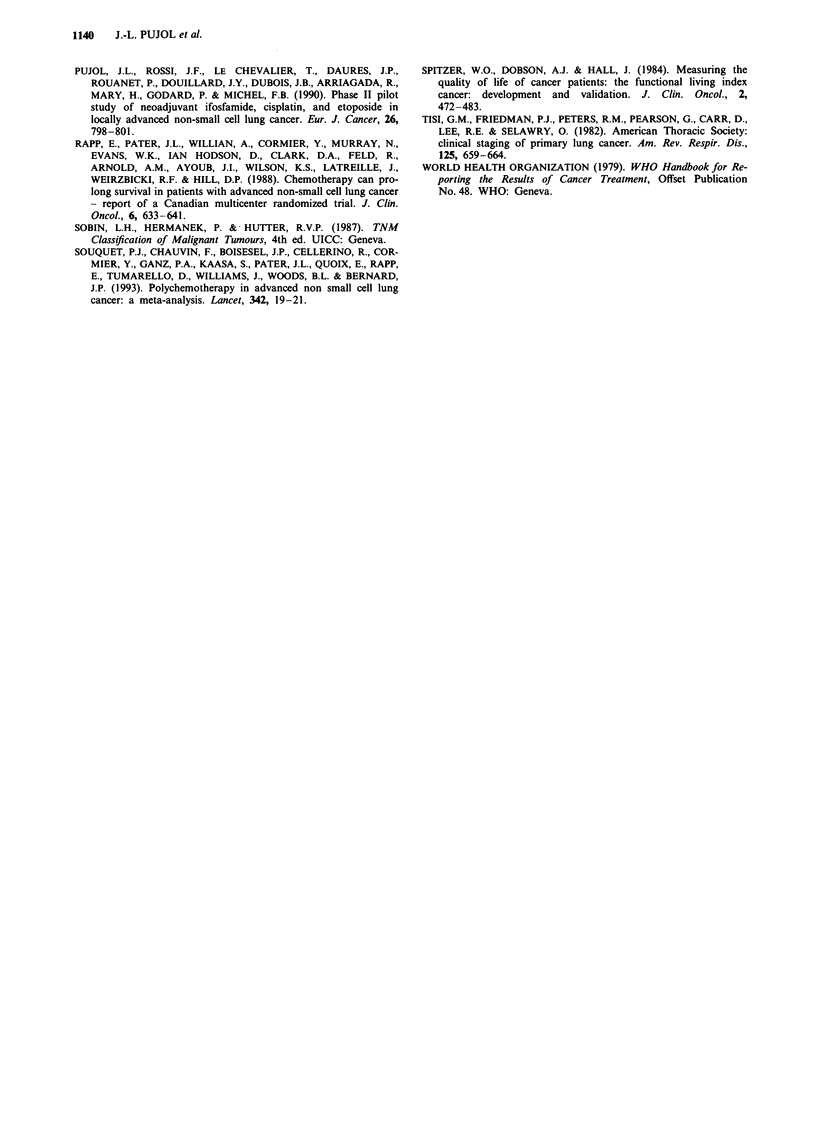# Phase II study of nitrosourea fotemustine as single-drug chemotherapy in poor-prognosis non-small-cell lung cancer.

**DOI:** 10.1038/bjc.1994.223

**Published:** 1994-06

**Authors:** J. L. Pujol, A. Monnier, J. Berille, M. L. Cerrina, J. Y. Douillard, A. Rivière, A. Grandgirard, S. Gouva, J. P. Bizzari, T. Le Chevalier

**Affiliations:** Service des Maladies Respiratoires, Université de Montpellier, Hôpital Arnaud de Villeneuve, France.

## Abstract

A phase II study was designed to evaluate objective response rate and toxicity of fotemustine as single-drug chemotherapy in non-small-cell lung cancer. Eighty-seven patients with unresectable non-small-cell lung cancer took part in the study. Seventy-seven were evaluable for response. Of these, 60% had received prior chemotherapy and 74% had metastatic disease. Moreover, 22 patients had central nervous system metastases (of whom 12 were evaluable for this site). Treatment consisted of fotemustine 100 mg m-2 administered on days 1 and 8 followed by a 5 week rest period. Afterwards, responding or stabilised patients received fotemustine 100 mg m-2 every 3 weeks as a maintenance therapy. Toxicity and quality of life were recorded during therapy. Thirteen patients (17%; 95% CI 9-25%) had an objective response (11% for pretreated, 26% for non-pretreated) with a median duration of 22 weeks (range 7-41 weeks). Two objective responses were observed among the 12 patients with evaluable brain metastases. No response was observed among the 14 patients with adenocarcinoma. Haematological, gastrointestinal, hepatic and renal toxicities were mild to moderate and manageable. The most frequent biological adverse reactions were delayed thrombocytopenia and neutropenia. Quality of life did not significantly decrease during the first 6 treatment weeks. Moreover, it remained stable during the study period in patients with response or stabilisation, whereas it significantly decreased in patients who experienced progression of the disease. Fotemustine is feasible for single-drug chemotherapy in non-small-cell lung cancer even though poor prognostic variables such as brain metastases are present. It can be administered on an outpatient basis and toxicity is moderate and manageable. Thus, fotemustine can be considered as a putative drug in further combinations.


Br. J. Cancer (1994), 69, 1136–1140 © Macmillan Press Ltd., 1994

### Phase II study of nitrosourea fotemustine as single-drug chemotherapy in poor-prognosis non-small-cell lung cancer

J.-L. Pujol[1], A. Monnier[2], J. Berille[3], M.-L. Cerrina[4], J.-Y. Douillard[5], A. Rivière[6], A. Grandgirard[2], S. Gouva[7], J.-P. Bizzari[3] & T. Le Chevalier[8]

[1]*Service des Maladies Respiratoires, Université de Montpellier, Hôpital Arnaud de Villeneuve, 34295 Montpellier Cedex;* [2]*Service d'Oncologie Médicale, Hopital de Montbéliard;* [3]*Institut de Recherches Internationales Servier, 6, place des Pléiades, 92415 Courbevoie;* [4]*Service de pneumologie du Professeur Duroux, Hôpital Antoine Beclère, Clamart;* [5]*Centre Anti-Cancéreux, René Gauducheau, Nantes;* [6]*Centre Anti-cancéreux, François Baclesse, Caen;* [7]*Service de pneumologie du professeur Clavier, Brest;* [8]*Institut Gustave Roussy, Villejuif, France.*

A phase II study was designed to evaluate objective response rate and toxicity of fotemustine as single-drug chemotherapy in non-small-cell lung cancer. Eighty-seven patients with unresectable non-small-cell lung cancer took part in the study. Seventy-seven were evaluable for response. Of these, 60% had received prior chemotherapy and 74% had metastatic disease. Moreover, 22 patients had central nervous system metastases (of whom 12 were evaluable for this site). Treatment consisted of fotemustine 100 mg m$^{-2}$ administered on days 1 and 8 followed by a 5 week rest period. Afterwards, responding or stabilised patients received fotemustine 100 mg m$^{-2}$ every 3 weeks as a maintenance therapy. Toxicity and quality of life were recorded during therapy. Thirteen patients (17%; 95% CI 9–25%) had an objective response (11% for pretreated, 26% for non-pretreated) with a median duration of 22 weeks (range 7–41 weeks). Two objective responses were observed among the 12 patients with evaluable brain metastases. No response was observed among the 14 patients with adenocarcinoma. Haematological, gastrointestinal, hepatic and renal toxicities were mild to moderate and manageable. The most frequent biological adverse reactons were delayed thrombocytopenia and neutropenia. Quality of life did not significantly decrease during the first 6 treatment weeks. Moreover, it remained stable during the study period in patients with response or stabilisation, whereas it significantly decreased in patients who experienced progression of the disease. Fotemustine is feasible for single-drug chemotherapy in non-small-cell lung cancer even though poor prognostic variables such as brain metastases are present. It can be administered on an outpatient basis and toxicity is moderate and manageable. Thus, fotemustine can be considered as a putative drug in further combinations.

Chemotherapy is still an experimental treatment in a majority of patients with non-small-cell lung cancer (NSCLC) (Hansen, 1988). Combinations of cisplatin and vinca alkaloids induce a survival improvement in unresectable NSCLC when compared with best supportive care. However, the ratio of the benefit in duration of survival to the benefit in quality of life (QL) might be suboptimal owing to the high frequency of toxic events induced by cisplatin-based regimens. Among NSCLC patients, those who relapse after first-line chemotherapy or those presenting central nervous system metastases are considered to have poor prognosis and are usually ineligible for cisplatin-based therapy. Thus, a search for new drugs is required in an attempt to obtain a survival advantage without inacceptable toxicity, particularly in patients with poor-prognosis NSCLC.

Fotemustine, a new amino acid phosphonate derivative of the nitrosourea group, has been recently introduced in the treatment of human solid malignancies (Avril *et al.*, 1990; Jacquillat *et al.*, 1990*a*). This cytotoxic drug induced a 24% objective response (OR) rate in a phase II study of fotemustine as a single-drug therapy in disseminated malignant melanoma (Jacquillat *et al.*, 1990*a*), including patients with brain metastases (Jacquillat *et al.*, 1990*b*). This drug has also been reported to be active in primary brain tumours (Frenay *et al.*, 1991). In a previous phase II study, we tested fotemustine in the treatment of squamous cell carcinoma using the 100 mg m$^{-2}$ dosage on a day 1, 8 and 15 regimen followed by a 5 week rest period, with maintenance therapy thereafter consisting of a course of 100 mg m$^{-2}$ every 3 weeks (Le Chevalier *et al.*, 1989). An OR rate of 12% was observed but the treatment was associated with a 30% grade IV thrombopenia toxicity. It has been observed, in a more recent study of fotemustine 100 mg m$^{-2}$ in melanoma, that the day 1, day 8 schedule is well tolerated with a similar efficacy (Avril *et al.*, 1990).

Correspondence: J.-L. Pujol.
Received 8 November 1993; and in revised form 18 January 1994.

We report herein a French multicentre phase II study on fotemustine on day 1 and day 8 in patients with poor-prognosis advanced NSCLC.

### Patients and methods

### *Eligibility criteria*

Patients of both sexes with histologically proven and unresectable NSCLC were entered. Inclusion criteria were: age below 75 years; Karnofsky index $\geqslant$60%; baseline leucocytes 4,000 $\mu$l$^{-1}$ or more, neutrophils 2,000 $\mu$l$^{-1}$ or more and platelets 150,000 $\mu$l$^{-1}$ or more; bilirubin, alkaline phosphatase, serum glutamic oxaloacetic transaminase (SGOT) and serum glutamic pyruvic transaminase (SGPT) lower than twice the normal upper limits; measurable or evaluable disease; and written informed consent. Staging was carried out by exhaustive procedure according to the fourth edition of the UICC-TNM classification and the American Thoracic Society map of regional pulmonary nodes (Tisi *et al.*, 1982; Sobin *et al.*, 1987). Patients presenting central nervous system metastases were eligible, whereas patients for whom bone metastases were the only evaluable lesions were not included. Only evolutive lesions (i.e. lesions for which a progression before inclusion could be documented) were taken as indicator lesions for chemotherapy response measurement. Patients who had undergone previous chemotherapy and/or radiotherapy treatment were eligible if the disease was considered as evolutive and if 4 weeks or more had elapsed between the end of the first treatment and entrance into this study. This delay was prolonged to 8 weeks if haematopoietic sites had been included in the radiation fields.

### *Treatment schedule and study design*

Fotemustine was provided by the Servier International Research Institute. The compound was administered over a

60 min intravenous infusion in a 5% glucose solution, which was protected from light. Induction treatment consisted of fotemustine 100 mg $m^{-2}$ administered on days 1 and 8 followed by a 5 week rest period. During this period, on days 8, 22 and 36 toxic events were recorded according to the World Health Organization (1979) scale. In particular, the following parameters were monitored: clinical assessment of overall toxicity, QL index, blood cell count, hepatic and renal functions. On day 43, the above-mentioned parameters were recorded again and tumour response was evaluated by measuring the indicator lesions. Responses were carefully reviewed by a panel of investigators.

Afterwards, patients for whom an OR was demonstrated and patients with stable disease underwent a maintenance chemotherapy consisting of fotemustine 100 mg $m^{-2}$ every 3 weeks until progression of the disease or grade III–IV toxicity (or both) occurred. Throughout the treatment, reduction in the administered fotemustine dose was decided on the basis of both haematological toxicity and hepatic function tests.

A QL index (Spitzer *et al.*, 1984) was recorded at the time of inclusion and afterwards on days 8, 22, 36 and 43. In addition, we assessed five important somatic variables: appetite, body weight, pain, sleep and fatigue. At each reassessment, the variables were scored as follows: −1, 0 and +1 for impairment, stabilisation and improvement respectively. These variables were included in an original somatic scale consisting of the sum of the five scores.

### *Tumour response measurement*

Tumour response was assessed using the WHO response criteria (World Health Organization, 1979). Indicator lesions were clearly defined at the time of inclusion and the technique used for measurement was also defined (usually CT scan). A complete response (CR) was defined as the complete disappearance of all lesions; a partial response (PR) was defined as ⩾50% reduction in the product of the two longest perpendicular diameters of the indicator lesion for measurable tumours, or an improvement ⩾50% for evaluable-only indicator lesions. A minor response (MR) was defined as a 25–49% decrease in the measurable indicator lesion in the product of the two longest perpendicular diameters of the indicator lesions, whereas stable disease (SD) was defined as a less than 25% reduction and a less than 25% increase in this product. Both minor MRs and SD were pooled into a stable group (ST). All responses and stabilisation had to be confirmed 4 weeks later without appearance of new lesions. Finally, progressive disease (PD) was defined as a ⩾25% increase in this product or appearance of new lesions.

### *Statistics*

The two-step Gehan method was used in order to determine the number of patients required to evaluate the anti-tumour activity with a $\beta$-risk ⩽5% (Gehan *et al.*, 1961). The anti-tumour activity was expressed as the OR rate (CR + PR) and 95% confidence interval (CI). Comparisons of the incidence of toxic events or response rates between pretreated patients and others were made using the chi-square test. Consecutive assessments of the QL index were analysed using the two-way analysis of variance and the Newman-Keuls test; this analysis was done for the entire population and for sub-groups defined by the response classification. Survival was defined as the time from the date of induction to the date of death. Probability of survival was estimated by the Kaplan–Meier method (Kaplan *et al.*, 1958). Complete response duration was defined as time from complete response to the time of progression and partial response duration from first course of treatment to the time of progression.

### Results

### *Patients' characteristics*

Between June 1989 and April 1991, 87 patients from seven institutions were enrolled (Table I). Of these 77 (88.5%) were evaluable for response. Ten patients were excluded: two because of protocol violations, four because of early deaths unrelated to the disease, three because of early deaths related to cancer and one because of loss to follow-up. One 79-year-old patient was included in the analysis despite a minor protocol violation. Among the 77 evaluable patients, 57 (74%) had metastatic disease; 55 had more than one metastatic site and 22 (29%) had brain metastases. Among these 22 patients, 12 were evaluable and 10 had received a prior radiotherapy. As shown in Table I, most of the patients were pretreated with first-line chemotherapy.

**Table I** Patient characteristics

| | |
|---|---|
| Number | 77 |
| Sex | 66M/11F |
| Median age (range) | 60 (33–79) |
| Histology | |
| SQC | 51 |
| Ad | 14 |
| LCC | 10 |
| BaC | 1 |
| Adenosquamous | 1 |
| Karnofsky index (KI) (%) | |
| 100 | 4 (5)[a] |
| 90 | 5 (6) |
| 80 | 32 (42) |
| ⩽70 | 36 (47) |
| Median KI for the whole population | 80% |
| Stage of disease | |
| I and II | 4 (5) |
| III | 16 (21) |
| IV | 57 (74) |
| Previous treatment | |
| None | 19 (25) |
| CT ± surgery ± RT | 46 (60) |
| Other than CT | 21 (15) |

[a]Four patients had asymptomatic metastatic disease. Abbreviations: Ad, adenocarcinoma; SQC, squamous cell carcinoma; LCC, large-cell carcinoma; CT, chemotherapy; RT, radiotherapy; BaC, bronchioloalveolar carcinoma.

### *Response*

Among the 77 evaluable patients, an objective response was observed in 13 (17%, 95% CI 9–25%), consisting of one CR and 12 PRs, and their characteristics are listed in Table II. Twenty-one (27%) had a stabilisation (1 MR and 20 SD) and 43 (56%) progressed. Median duration of response was 22 weeks (range 7–41 weeks). Of 46 patients who had received previous chemotherapy, five responded to fotemustine, giving an OR rate of 11% (95% CI 2–20%), whereas 8 out of 31 non-pretreated patients had a response (OR 26%, CI 10–41%). However, this difference did not reach a statistical significance ($\chi^2 = 2.94$). Among the 12 patients with evaluable brain metastases, we observed two responses (one complete lasting 11 weeks and one partial lasting 23 weeks). Twelve patients had liver metastases, but only ten of them had an evaluable lesion; among these three complete regressions were recorded. Forty-six patients received a maintenance therapy with an average of three fotemustine courses (range 1–13); this population included 13 responding patients and 21 stable patients. Twelve patients who had a symptomatic response and experienced no evidence of toxic effect also received maintenance therapy, although tumour measurement at week 5 demonstrated progression.

### *Toxicity*

Haematological toxicity was moderate and manageable (Table III). It consisted mainly of delayed thrombocytopenia

**Table II** Characteristics of responding patients

| | |
|---|---|
| Number | 13 |
| Sex | 13M |
| Median age (range) | 61 (38–73) |
| Histology | |
| SQC | 11 |
| LCC | 2 |
| Karnofsky index (KI) (%) | |
| 100 | 1 |
| 90 | 1 |
| 80 | 9 |
| ⩽70 | 2 |
| Median KI for the whole population | 80% |
| Stage of disease | |
| I, II and III | 15% |
| IV | 85% |
| Previous treatment | |
| None | 4 |
| CT ± surgery ± RT | 5 |
| Other than CT | 4 |
| Responding indicator lesions in metastatic sites (PR/CR/no. of patients with evaluable indicator lesions) | |
| Liver | 0/3/10 |
| Central nervous system | 1/1/12 |
| Kidney | 1/0/1 |

**Table III** Toxicity of fotemustine (% of affected patients)

| | WHO grade | | | | | |
|---|---|---|---|---|---|---|
| *Toxicity* | *0* | *I* | *II* | *III* | *IV* | *NE* |
| Haemoglobin | 44 | 24 | 18 | 5 | 3 | 6 |
| White cell count | 51 | 15 | 9 | 8 | 1 | 16 |
| Neutrophil count | 57 | 12 | 5 | 7 | 3 | 16 |
| Platelet | 51 | 13 | 7 | 11 | 7 | 11 |
| SGOT | 80 | 7 | 2 | 1 | 0 | 10 |
| SGPT | 77 | 13 | 0 | 0 | 0 | 10 |
| Bilirubin | 79 | 6 | 1 | 0 | 0 | 14 |
| Alkaline phosphatase | 63 | 18 | 4.5 | 4.5 | | 10 |
| Creatinine | 83 | 9 | 0 | 0 | 0 | 8 |
| Nausea, vomiting | 79 | 8 | 9 | 3.7 | 0 | 0 |

Abbreviations: SGPT, serum glutamic pyruvic transaminase; SGOT, serum glutamic oxaloacetic transaminase; NE, not evaluable.

(median nadir day 36) and, to a minor extent, neutropenia. The incidence of grade III–IV thrombopenia was higher in pretreated patients than in non-pretreated ones (8% *vs* 26%, $P<0.05$), whereas neutropenia and leucopenia did not significantly differ. We observed no neutropenia-induced fever. Three patients required both red blood cell and platelet transfusions.

Gastrointestinal toxicity was moderate in as much as grade III nausea and vomiting was only reported in 3.7% of 129 analysed courses; and two patients had diarrhoea. Hepatic toxicity resulted in a moderate and reversible increase in SGPT, SGOT, bilirubin and alkaline phosphatase levels, and none of the patients experienced a grade IV toxicity or a clinical hepatic toxicity. Moreover, there was only one patient with a grade III increase in SGOT. In two cases biological adverse effects might possibly be related to treatment, whereas in the other instances liver metastases and/or pretreatment might be responsible for these biological modifications. There was no severe modification of renal function.

### *Survival*

Median survival of the whole population was 20 weeks (range 2–111 + weeks; Figure 1). It did not significantly differ when pretreated patient survival was compared with other patient survival [median survival of 20 weeks (range 2–91 + weeks) *vs* 19 weeks (range 2–111) weeks respectively]. Moreover, the subgroup of patients with brain metastases had an identical survival rate. The probability of survival at 9 and 12 months was 29 and 14% respectively. At the time of reporting, seven patients had survived for more than 1 year. Their mean age was 57 years (range 38–73). All had a stage IV squamous cell carcinoma with a Karnofsky index of 80%. Four had received chemotherapy prior to entering this study and four had received brain radiation therapy for brain metastases. In these seven patients, induction chemotherapy using fotemustine resulted in a partial response in five cases and stabilisation in one, whereas the disease progressed in the remaining patient.

### *Quality of life*

QL index was reliable and fully analysed in 54 patients. The mean QL of the whole population decreased slightly during treatment when comparing day 0 with day 43. However, the relation of QL versus time was not significant (Figure 2; $P=0.09$). QL index remained stable during the study period for patients with response or stabilisation, whereas it significantly decreased ($P<0.001$) in patients who experienced progression of the disease. The somatic scale was fully assessed in 60 patients (69%). This decreased significantly during the study duration ($P=0.001$). However, in patients with response or stable disease the somatic scale did not vary according to time (Figure 3). Mean expanded on-study time was 97 days (range 3–385 days). Only five patients were admitted for treatment of a toxic event related to fotemustine. Twenty-nine patients (33%) were never hospitalised, even for fotemustine treatment. This subgroup and the entire population had a similar duration of on-study time (90 and 97 days respectively).

**Figure 1** Overall survival (whole population).

**Figure 2** Quality of life index (■, $P=0.09$) and somatic scale (●, $P=0.001$) during the induction treatment in the entire population. Statistics using analysis of variance. Mean and standard error of the mean are presented.

**Figure 3** Somatic scale during the induction treatment according to response. Mean somatic evaluations are presented. Analyses of variance showed a significant decrease in the progression group (O---O, $P<0.001$), whereas the variations were non-significant in both responding (□—□) and stable (◇—◇) groups ($P=0.40$).

### Discussion

Chemotherapy of NSCLC is still a subject of controversy (Idhe, 1992). The results of single-drug chemotherapy for this disease have been extensively reviewed (Bunn, 1991; Donnadieu *et al.*, 1991). Few antineoplastic agents allow patients to achieve more than a 15% OR rate: of these, cisplatin, ifosfamide, mitomycin, vindesine, vinblastine and, more recently, vinorelbine (Navelbine) are the most frequently used drugs in combination. A recent meta-analysis of the randomised trials comparing chemotherapy and best supportive care in unresectable NSCLC demonstrated that chemotherapy significantly prolongs survival. However, the survival benefit is probably of modest duration (7–15 weeks), a fact that accounts for the difficulty in demonstrating a significant difference in small trials. The QL in patients who undergo chemotherapy has been, until now, very difficult to report, and it is not clear whether or not QL is better in chemotherapy-treated patients (Rapp *et al.*, 1988; Souquet *et al.*, 1993).

The benefit obtained by the use of chemotherapy in NSCLC is probably higher in locally advanced disease than in extensive disease (Pujol *et al.*, 1990; Donnadieu *et al.*, 1991). Thus, in the setting of locally advanced NSCLC, the main goal is to improve the rate of cure by including chemotherapy in combined-modality treatments. On the other hand, metastatic NSCLC is mainly an incurable disease for which chemotherapy must reach the best therapeutic index, i.e. antineoplastic activity without unacceptable toxicity. The use of cisplatin-based combinations is unfortunately limited by the high incidence of digestive toxicity and by the ineligibility of patients with poor Karnofsky index and/or renal dysfunction. Thus, for these patients other treatment modalities must be considered. Finally, it is necessary to propose treatment to patients with an acceptable Karnofsky index but poor prognosis, i.e. patients who need a second-line treatment or patients presenting with brain metastases. For these patients the choice should be a compromise between potential benefit in survival and QL and decrease in hospitalisation and cost. However, until now, most of the new drugs have been investigated as first-line treatment, whereas others are inefficient as second-line therapy. Thus, new active drugs in patients with poor prognostic factors are needed because their treatment is a frequent problem in current practice.

The present study demonstrates the feasibility of a chemotherapy regimen consisting of fotemustine 100 mg $m^{-2}$ administered on a day 1 and 8 schedule. It is noteworthy that the inclusion criteria of this trial matched the characteristics of the patient population admitted into our institutions, in particular pretreated patients or patients with central nervous system metastases. Both features, usually excluded from conventional chemotherapy trials, were eligible in this study and the results in these subgroups were good even though they were slightly lower than in other patients. Overall toxicity can be considered as mild to moderate and manageable. The low haematological toxicity, even in pretreated patients, and the low gastrointestinal, hepatic and renal toxicities make it possible to propose fotemustine as a treatment for poor-prognosis NSCLC even though renal function is impaired. Of interest, there was no major modification of the QL index during the study and the somatic scale proposed by this study only decreased in patients who progressed during the treatment. Thus, fotemustine seems not to impair the QL.

To be considered as active, an antineoplastic drug must produce a response rate >15% (Hansen, 1988). Both response rate and response duration observed in this study were over this limit. Fotemustine had, therefore, a good therapeutic index in as much as the 17% OR rate is associated with neither life-threatening toxic events nor impairment of the QL and is an applicable treatment in poor-prognosis NSCLC. Our results deserve further evaluation of fotemustine in combination with other active drugs in NSCLC.

### References

AVRIL, M.F., BONNETERRE, J., DELAUNAY, M., GROSSHAMS, E., FUMOLEAU, P., ISRAEL, L., BUGAT, R., NAMER, M., CUPISSOL, D., KERBRAT, P., MONTCUQUET, P., ARCAUTE, V. & BIZZARI, J.P. (1990). Combination chemotherapy of dacarbazine and fotemustine in disseminated malignant melanoma. *Cancer Chemother. Pharmacol.*, **27**, 81–84.

BUNN, P.A. (1991). The role of systemic chemotherapy in non-small cell lung cancer. In *Current Topics in Lung Cancer*, Veronesi, U. (ed.) pp. 33–43. Springer: Berlin.

DONNADIEU, N., PAESMANS, M. & SCULIER, J.P. (1991). Chemotherapy of non-small cell bronchial cancers. Meta-analysis of the literature as a function of the extent of the disease. *Rev. Mal. Respir.*, **8**, 197–204.

FRENAY, M., GIROUX, B., KHOURY, S., DERLON, J.M. & NAMER, M. (1991). Phase II study of fotemustine in recurrent supratentorial malignant gliomas. *Eur. J. Cancer*, **27**, 852–856.

GEHAN, E.A. (1961). The determination of the number of patients required in a preliminary and follow up trial of a new chemotherapeutic agent. *J. Chron. Dis.*, **13**, 346–353.

HANSEN, H.H. (1988). Lung cancer. In *Cancer: Chemotherapy and Biological Response Modifiers*, Annual 10, Pinedo, H.M., Longo, D.L. and Chabner, B.A. (eds) pp. 222–240. Elsevier: Amsterdam.

IDHE, D.C. (1992). Chemotherapy of lung cancer. *N. Engl. J. Med.*, **327**, 1434–1441.

JACQUILLAT, C., KHAYAT, D., BANZET, P., WEIL, M., AVRIL, M.F., FUMOLEAU, P., NAMER, M., BONNETERRE, J., KERBRAT, P., BONERANDI, J.J., BUGAT, R., MONTCUQUET, P., AUDHUY, B., CUPISSOL, D., LAUVIN, R., GROSSHANS, E., VILMER, C., PRACHE, C. & BIZZARI, J.P. (1990*a*). Chemotherapy by fotemustine in cerebral metastases of disseminated malignant melanoma. *Cancer Chemother. Pharmacol.*, **25**, 263–266.

JACQUILLAT, C., KHAYAT, D., BANZET, P., WEIL, M., FUMOLEAU, P., AVRIL, M.F., NAMER, M., BONNETERRE, J., KERBRAT, P., BONERANDI, J.J., BUGAT, R., MONTCUQUET, P., CUPISSOL, D., LAUVIN, R., VILMER, C., PRACHE, C. & BIZZARI, J.P. (1990*b*). Final report of the French multicenter phase II study of nitrosourea fotemustine in 153 evaluable patients with disseminated malignant melanoma including patients with cerebral metastases. *Cancer*, **66**, 1873–1878.

KAPLAN, E.L. & MEIER, P. (1958). Nonparametric estimation from incomplete observations. *J. Am. Stat. Assoc.*, **53**, 457–481.

LE CHEVALIER, T., ZABBE, C., GOUVA, S., CERRINA, M.L., QUOIX, E., RIVIERE, A., BERTHAUD, P., PRACHE, C. & BERILLE, J. (1989). Phase II multicentre study of the nitrosourea fotemustine in inoperable squamous cell lung carcinoma. *Eur. J. Cancer Clin. Oncol.*, **25**, 1651–1652.

PUJOL, J.L., ROSSI, J.F., LE CHEVALIER, T., DAURES, J.P., ROUANET, P., DOUILLARD, J.Y., DUBOIS, J.B., ARRIAGADA, R., MARY, H., GODARD, P. & MICHEL, F.B. (1990). Phase II pilot study of neoadjuvant ifosfamide, cisplatin, and etoposide in locally advanced non-small cell lung cancer. *Eur. J. Cancer*, **26**, 798-801.

RAPP, E., PATER, J.L., WILLIAN, A., CORMIER, Y., MURRAY, N., EVANS, W.K., IAN HODSON, D., CLARK, D.A., FELD, R., ARNOLD, A.M., AYOUB, J.I., WILSON, K.S., LATREILLE, J., WEIRZBICKI, R.F. & HILL, D.P. (1988). Chemotherapy can prolong survival in patients with advanced non-small cell lung cancer – report of a Canadian multicenter randomized trial. *J. Clin. Oncol.*, **6**, 633-641.

SOBIN, L.H., HERMANEK, P. & HUTTER, R.V.P. (1987). *TNM Classification of Malignant Tumours*, 4th ed. UICC: Geneva.

SOUQUET, P.J., CHAUVIN, F., BOISESEL, J.P., CELLERINO, R., CORMIER, Y., GANZ, P.A., KAASA, S., PATER, J.L., QUOIX, E., RAPP, E., TUMARELLO, D., WILLIAMS, J., WOODS, B.L. & BERNARD, J.P. (1993). Polychemotherapy in advanced non small cell lung cancer: a meta-analysis. *Lancet*, **342**, 19-21.

SPITZER, W.O., DOBSON, A.J. & HALL, J. (1984). Measuring the quality of life of cancer patients: the functional living index cancer: development and validation. *J. Clin. Oncol.*, **2**, 472-483.

TISI, G.M., FRIEDMAN, P.J., PETERS, R.M., PEARSON, G., CARR, D., LEE, R.E. & SELAWRY, O. (1982). American Thoracic Society: clinical staging of primary lung cancer. *Am. Rev. Respir. Dis.*, **125**, 659-664.

WORLD HEALTH ORGANIZATION (1979). *WHO Handbook for Reporting the Results of Cancer Treatment*, Offset Publication No. 48. WHO: Geneva.